# Pulmonary Embolism in Patients with Lung Cancer: Incidence and Performance of Prognostic Markers

**DOI:** 10.3390/cancers18111838

**Published:** 2026-06-04

**Authors:** Francesco Tannura, Andrea Sbrana, Antonio Chella, Francesco Pistelli, Laura Carrozzi, Alessandro Celi, Roberta Pancani

**Affiliations:** 1 Department of Surgical, Medical and Molecular Pathology, and Critical Care, University of Pisa, 56126 Pisa, Italy; f.tannura@studenti.unipi.it (F.T.); francesco.pistelli@unipi.it (F.P.); laura.carrozzi@unipi.it (L.C.); 2 Respiratory Unit, Cardio-Thoracic and Vascular Department, University Hospital of Pisa, 56126 Pisa, Italy; andrea.sbrana@ao-pisa.toscana.it (A.S.); a.chella@ao-pisa.toscana.it (A.C.); roberta.pancani@ao-pisa.toscana.it (R.P.)

**Keywords:** pulmonary embolism, lung cancer, prognostic risk assessment, Khorana Risk Score

## Abstract

Background: The incidence of cancer-associated thromboembolism has been extensively investigated, but mostly in heterogeneous cancer populations. Prognostic risk assessment is crucial in pulmonary embolism, but the accuracy of the commonly used tools remains uncertain in patients with cancer. Methods: We retrospectively included outpatients with consecutive lung cancer attending the Pulmonary Unit in Pisa from July 2019 to June 2021. The study population was the subgroup of patients who developed at least one episode of pulmonary embolism. For all patients, clinical data within 72 h of pulmonary embolism diagnosis, Khorana Risk Score, and overall survival time were collected. Results: A total of 512 patients with lung cancer attended the clinic; 40 patients developed pulmonary embolism (cumulative incidence of 7.81%). Eight patients (20%) died within a month, and twenty-two patients (55%) died within 6 months. Troponin, N-terminal pro-brain-type natriuretic peptide and shock index were significantly different between survivors and non-survivors (*p* < 0.05). Pulmonary artery diameter and the right to left ventricle index were not significantly different between survivors and non-survivors. Patients’ survival significantly decreased with the increase in Khorana Risk Score. Conclusions: Compared to previous studies, a higher incidence of pulmonary embolism in lung cancer was detected by our study. The prognosis of patients with lung cancer with pulmonary embolism seemed to be influenced more by the natural history of cancer than by the severity of pulmonary embolism. Khorana Risk Score might be considered as a prognostic tool in patients with lung cancer and may be used in the prognostic work-up for lung cancer-associated thromboembolism after a prospective validation.

## Simple Summary

Cancer is frequently associated with venous thromboembolism. Most studies on cancer-associated thrombosis are based on mixed cohorts including different cancer types. Moreover, the prognostic factors for venous thromboembolism have not been specifically assessed in cancer-associated thrombosis. In a population of outpatients with lung cancer, we detected an incidence of 7.81% over two years, slightly higher than previously reported in comparable subpopulations. Cancer stage and, somewhat unexpectedly, the Khorana Risk Score were prognostic markers more reliable than other commonly used markers, including the ratio between the right and left ventricles and the pulmonary artery diameter. Our data reduce the impact of the prognostic indices that have been validated in the general population.

## 1. Introduction

Venous thromboembolism (VTE) is the third most frequent acute cardiovascular syndrome [1,2], and it is associated with a reduced survival and important health-care costs [3]. There are many risk factors that can promote the onset of VTE, and active cancer is one of the most significant [1,4]. The pathogenesis of cancer-associated thromboembolism (CAT) is complex and not fully understood. It is known, for example, that cancer cells induce the synthesis of angiogenetic factors that have concomitant procoagulant activity, such as tissue factor. Furthermore, cancer activates blood and vascular cells, thus increasing their prothrombotic properties. Finally, anticancer drugs exert procoagulant activities, largely through endothelial activation [5].

Patients with lung cancer present a state of hypercoagulability, even in the absence of clinically manifest thrombosis; several mechanisms contribute to this state of hypercoagulability, including the release of pro-thrombotic extracellular vesicles into the circulation [6] and, on the other hand, the reduction in the anticoagulant activity of certain molecules, such as heparinase [7]. CAT remains a major health problem due to its impact on morbidity, mortality and excess use of economic resources [8,9]. Indeed, thromboembolism is the second most important cause of death after disease progression in patients with cancer receiving chemotherapy [10]. The incidence of CAT has been extensively investigated. It is known that it may vary depending on tumor site, histotype, disease state, therapy and other individual factors [11]; however, the literature data usually lump together patients with tumors in different organs, and the study populations are extremely heterogeneous. In the era of precision medicine, it is important to collect data in homogeneous populations, working towards increasingly personalized patient management. The prognostic risk assessment is a crucial step to identify patients with pulmonary thromboembolism (PE) at risk of early death and to choose the most adequate treatment [1,2]; current European guidelines identify the presence of shock, pulmonary embolism severity index (PESI) score or simplified PESI score (sPESI) [12,13], right ventricle strain using CT scan or echocardiography [14,15] and t-troponin level as the relevant markers to stratify prognosis [16]. Whether this panel, validated mostly in patients without cancer, is also appropriate for patients with cancer is not completely clear [17]; specifically, it is important to note that the PESI and sPESI scores were validated in a population where patients with cancer represented less than 20% of the total [18].

The main research questions of this study were to assess the cumulative incidence of PE in a population of patients with lung cancer and to evaluate the performance of validated prognostic tools for PE in this group of patients with lung cancer.

## 2. Patients and Methods

We retrospectively included outpatients with consecutive lung cancer attending the Pulmonary Oncology service affiliated with the Pulmonary Unit in Pisa (Italy) from July 2019 to June 2021.

We used electronic records to identify the subgroup of patients with consecutive lung cancer who developed at least one episode of PE diagnosed by computed tomography-pulmonary angiography (CTPA) according to standard methods [1,2]. No exclusion criteria were defined.

Within 72 h from the PE diagnosis, the following data were collected:

- Anthropometric generalities of subject (sex, age, height, weight, body mass index (BMI), date of death if any);
- Medical history (previous episode(s) of VTE, presence of chronic heart and/or lung disease);
- Lung cancer characterization;
- Histotype (in detail, we distinguished “adenocarcinomas” from “non-adenocarcinomas”);
- Staging, according to 2018 TNM methodology [19];
- Localization of PE based on CTPA (defined as “proximal” if there was involvement of the pulmonary artery and/or interlobar/lobar branches and “distal” if there was involvement from segmental branches onward);
- Khorana Risk Score (KRS) [20];
- Hemato-biochemical laboratory parameters (International normalized ratio (INR), creatinine, creatinine clearance (according to Cockcroft-Gault formula) [21], D-dimer, Fibrinogen, Cardiac T-troponin, N-terminal pro-brain-type natriuretic peptide (NT-pro-BNP);
- Arterial blood gas analysis parameters (pH, PaO_2_, PaCO_2_, bicarbonates, excess bases, oxygen saturation (SO_2_));
- Ongoing drug therapies, with specific attention to the recent course of radio- and/or chemotherapy, use of immunotherapy, molecular targeted drugs, antiplatelets or anticoagulants.

For prognostic risk assessment, according to the European Society of Cardiology (ESC)/European Respiratory Society (ERS) 2019 guidelines [1], we considered the following:

- T-troponin alteration;
- Right ventricle to left ventricle diameter ratio (RV/LV);
- PESI score;
- We also recorded shock index (SI), NT-proBNP and main pulmonary artery (PA) diameter on CTPA, although these parameters are not included in the ESC/ERS [1] algorithm for prognostic risk assessment.

RV/LV was classified as “positive” if >1 [1,22], while PA diameter was classified as “positive” when >29 mm and 27 mm for males and females, respectively [23]. CT scans were independently evaluated by two readers, a medical student and a pulmonary physician with more than 15 years of specific experience. For final analysis, however, in the rare event of discrepant measurements, the reading of the experienced physician was considered. In addition, the clinical course, including death, was recorded. Bleedings were classified as major or non-major and clinically significant according to the International Society of Thrombosis and Haemostasis (ISTH) guidelines [24].

The survival at 1 and 6 months from the diagnosis of PE was evaluated; in addition, a comparison between survivors and non-survivors was made considering both risk stratification parameters and tumor histotype.

Data were anonymized following the national regulation for personal data protection. The study was approved by the Comitato Etico Regionale per la Sperimentazione Clinica della Toscana—sezione Area Vasta NordOvest (approval number 23835, date of approval: 16 March 2023).

### Statistical Analysis

Statistical analyses were performed with Jamovi (version 2.3.6, 2022 for MacOS). The Shapiro test was used to verify the normality of data distribution. Continuous variables are shown as mean ± standard deviation (SD) or median (interquartile range) as appropriate; categorical variables are shown as numbers and percentages. For variables with two levels, we used the Student’s *t* test or the Mann–Whitney test; for variables with more than two levels, ANOVA was used. Inter-operator agreement was evaluated with Cohen’s Kappa test. Kaplan–Meier analyses were performed to graphically represent survival at 1 and 6 months; survivals were compared by log-rank analysis. Statistical significance was defined as a *p* value < 0.05.

## 3. Results

### 3.1. Study Population

During the study period, 512 patients with lung cancer attended the clinic; out of them, 40 patients (7.81%) developed PE.

The mean age was 72 years, with an equal distribution between males and females (50% each). A minority of patients had a history of cardiopulmonary disease, specifically chronic lung disease (22.5%) and heart failure (7.5%). Only one patient (2.5%) had a previous episode of VTE, and a single major bleeding event (2.5%) was recorded during the observation period. Most patients had metastatic lung cancer (65%) at PE diagnosis. The baseline characteristics of the study population and the full list of oncologic treatments are summarized in Table 1.

PE was distal in most cases (65%); 65% of patients had levels of troponin and/or NT-proBNP above the upper limit of normal of our laboratory. The PESI score was 124 ± 29.

Regarding radiological parameters, the Cohen’s Kappa test showed a good inter-operator agreement value (K = 0.77); the main PA diameter was 29 ± 4.92 mm, and the RV/LV index was 1.08 (0.93–1.23).

Arterial blood gas analysis showed a tendency toward respiratory alkalosis (pH 7.45 ± 0.04 and PaCO_2_ 34.5 ± 6.7 mmHg) (Table 2).

### 3.2. Prognostic Risk Assessment

**Figure 1:**
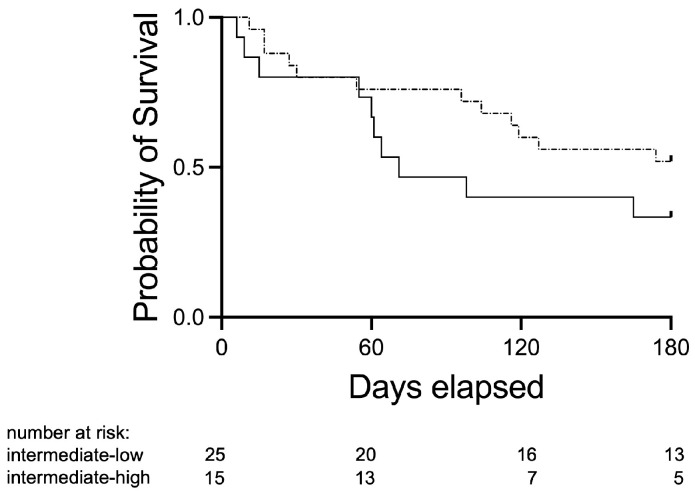
Kaplan–Meier survival curves. Dashed line: intermediate-low risk; solid line: intermediate-high risk.

According to the ESC/ERS 2019 guidelines [1], twenty-five patients (62.5%) were classified in the intermediate-low risk category and fifteen patients (37.5%) in the intermediate-high risk category. No significant difference in overall survival was observed between these two groups (*p* = 0.18, Figure 1).

### 3.3. Mortality and Survival Analysis

Eight patients (20%) died within 1 month and twenty-two patients (55%) died within 6 months after PE diagnosis.

Comparing survivors with non-survivors, T troponin and/or NT-proBNP and SI were significantly different (*p* = 0.04 at both 1 and 6 months and *p* = 0.01 at 1 month, respectively).

**Figure 2:**
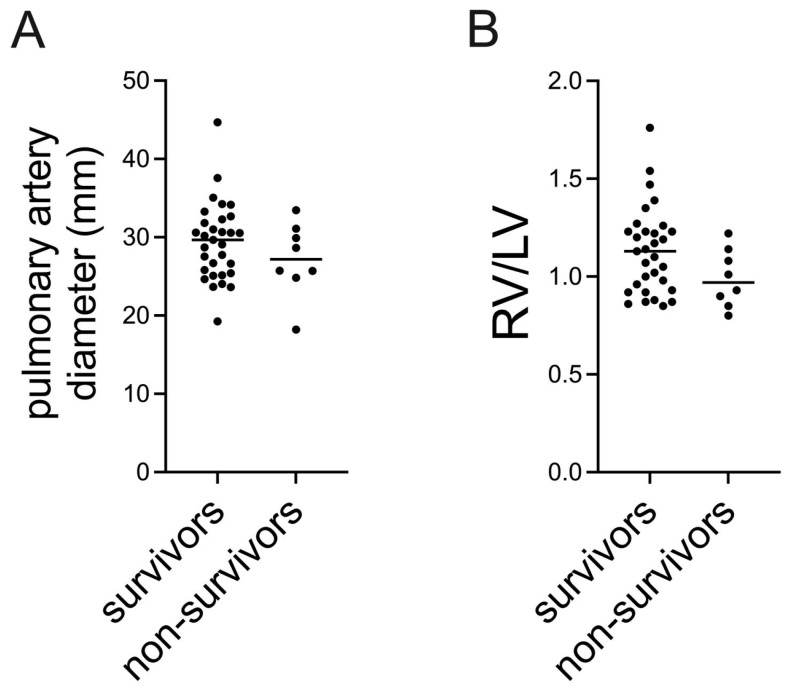
Main prognostic indices analysis. (**A**) Pulmonary artery diameter for survivors and non-survivors at one month. (**B**) RV/LV index for survivors and non-survivors at one month.

Main PA diameter, RV/LV ratio and PESI score were not significantly different between survivors and non-survivors at one month (Table 3, Figure 2A,B).

**Figure 3:**
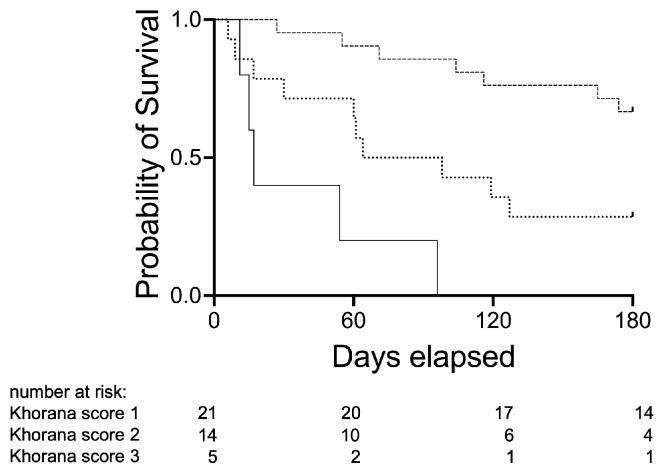
Survival analysis according to KRS. Dashed line: KRS 1; dotted line: KRS 2; solid line: KRS 3. *p* < 0.05 by log-rank analysis.

Finally, patients’ survival significantly decreased from KRS = 1 to KRS = 2 and, even more so, to KRS = 3 (*p* < 0.001, Figure 3), independent of lung cancer histotype; indeed, the same trend was found comparing lung adenocarcinoma subjects with non-adenocarcinoma subjects.

## 4. Discussion

The incidence of PE in our lung cancer population was 7.81% during the time observation period of the study, in line but with a slight upward trend compared to previous studies [25]; this may reflect the overall increase in PE incidence over the last decades [1]; an interesting, but yet speculative, hypothesis is that also the post-pandemic era may have contributed [26]. This increase could be due to multiple factors: on the one hand, recent years have seen an improvement in radiological techniques, particularly computed tomography, thus the rise in the detection rate; furthermore, novel therapeutic options have increased the life expectancy of this patients (indeed, aging is a risk factor for VTE [27]), at the same time exposing them to the potential prothrombotic effects of such therapies.

It is well known that many factors can promote the occurrence of venous thromboembolic events in patients with cancer, such as the presence of metastases and the time elapsed since the diagnosis of lung cancer; accordingly, the incidence in different study populations may vary slightly [28].

The second research question of our study involved the assessment of the performance of prognostic scores validated in the general population. We observed a relatively poor performance of such indexes. Indeed, not only the PESI score did not differ significantly between survivors and non-survivors (both at 1 and 6 months), but there was also no significant difference in overall survival among the commonly used prognostic risk assessment categories (Figure 1). Notably, we excluded the sPESI from the analysis because the presence of “active cancer” as a weighted criterion (assigning 1 point) automatically classifies all patients with lung cancer as “high risk” (sPESI ≥ 1) [13]. This approach may lead to an overestimation of risk and a high rate of false-positive classifications, potentially masking the actual clinical prognosis of our cohort.

Furthermore, neither PA diameter nor RV/LV had a negative impact on prognosis in this study population (Figure 2A and Figure 2B, respectively), although they are usually considered to be associated with poor short-term prognosis in subjects with PE [29].

As expected [16], the alteration of T-troponin and/or NT-proBNP negatively affected the survival of our study population, both at 1 month and 6 months; the shock index negatively affected only the 1-month survival of patients.

Some of the most interesting results came from the analysis of KRS. The KRS is a tool validated for predicting the risk of VTE for patients with cancer undergoing outpatient chemotherapy [20]; it includes body mass index (BMI), pre-chemotherapy platelet count, pre-chemotherapy leukocyte count, hemoglobin, and type of cancer. Although a few studies have also investigated the role of this score as a prognostic tool, the score is not commonly included in the prognostic work-up of CAT. In a recent study [30], a high KRS was a predictor of mortality in patients with lung cancer (HR 1.7); Vathiotis et al. [31] obtained similar results, and KRS proved to be an independent predictor of early mortality, rather than VTE, in patients with lung adenocarcinoma receiving first-line or adjuvant chemotherapy.

As shown by the Kaplan–Meier curve (Figure 3), subjects’ survival significantly decreased with the increasing of KRS; in a post hoc analysis, we obtained the same result by analyzing separately lung cancer adenocarcinomas and non-adenocarcinomas. In our opinion, a prospective validation of the KRS might lead to its inclusion in the prognostic work-up of CAT, especially for newly diagnosed patients with lung cancer, independent of histotype.

We are fully aware of the limitations of our study; this mostly pertains to the small sample size and its monocentric retrospective design, which implies the presence of missing values for several variables. Notably, our electronic health records lacked certain granular data, such as specific symptoms or concomitant deep vein thrombosis diagnosis; furthermore, a more comprehensive investigation is already ongoing to validate our results, also comparing diverse PE populations, in order to overcome the aforementioned limitations. On the other hand, this is one of the few studies that only analyzes a homogeneous population of patients with lung cancer, including all histotypes.

## 5. Conclusions

Our results suggest that the underlying neoplastic disease represents the most relevant prognostic driver, thus reducing the impact of the prognostic indices that have been validated in the general population. Therefore, in our opinion, the need to improve those indices is pressing.

KRS might be considered a prognostic tool in patients with lung cancer, independent of tumor histotype, and might be suggested in the prognostic work-up for CAT in lung cancer after an adequate prospective validation.

## Figures and Tables

**Table 1:** General characteristics, comorbidities, bleedings and oncologic treatments of the study population.

Subjects with PE *n* (%)	40 (100)
Sex *n* (%)	
Male	20 (50)
Female	20 (50)
Age (years, mean ± SD)	72 ± 8
BMI (mean ± SD)	25.7 ± 4.6
Chronic lung disease *n* (%)	9 (22.5)
Heart failure *n* (%)	3 (7.5)
Previous VTE *n* (%)	
Yes	1 (2.5)
No	39 (97.5)
Bleeding *n* (%)	
Major	1 (2.5)
Non-major	0 (0)
Metastasis *n* (%)	
Yes (M1)	26 (65)
No (M0)	14 (35)
Chemotherapy *n* (%)	
Yes	24 (60)
No	16 (40)
Immunotherapy *n* (%)	
Yes	19 (47.5)
No	21 (52.5)
Targeted therapy *n* (%)	
Yes	6 (15)
No	34 (85)
Khorana Risk Score *n* (%)	
0	0 (0)
1	21 (52.5)
2	14 (35)
3	5 (12.5)
4	0 (0)
5	0 (0)

**Table 2:** Survival time, radiological and arterial blood gas analysis parameters in patients with lung cancer with PE.

Subjects with PE *n* (%)	40 (100)
Site of PE *n* (%)	
Proximal	16 (35)
Distal	24 (65)
Death at 1 month *n* (%)	8 (20)
Death at 6 months *n* (%)	22 (55)
Increased troponin and or NT-proBNP levels *n* (%)	
Yes	26 (65)
No	14 (35)
Pulmonary artery diameter (mm, mean ± SD)	29 ± 5
RV/LV ratio index [median (interquartile range)]	1.08 (0.93–1.23)
PESI score (mean ± SD)	124 ± 28
pH (mean ± SD)	7.45 ± 0.04
PaO_2_ (mmHg, mean ± SD)	71.9 ± 15.8
PaCO_2_ (mmHg, mean ± SD)	34.5 ± 6.7
SO_2_ (%, mean ± SD)	94.2 ± 4.4

PE, pulmonary embolism; NT-proBNP, N-terminal pro-brain natriuretic peptide; RV, right ventricle; LV, left ventricle; PESI, pulmonary embolism severity index.

**Table 3:** Main prognostic indices comparison between 1-month survivors and non-survivors.

	1-Month Survivors	1-Month Non-Survivors	*p*
Pulmonary artery diameter (mm, mean ± SD)	29.4 ± 5.0	27.2 ± 4.7	0.26
Shock Index (mean ± SD)	0.66 ± 0.17	0.83 ± 0.33	**0.04**
PESI score (mean ± SD)	122 ± 19	124 ± 49	0.12
RV/LV index [median (interquartile range)]	1.13 (0.95–1.23)	0.97 (0.89–1.09)	0.10

## Data Availability

The raw data supporting the conclusions of this article will be made available by the authors on request.

## Abbreviations

The following abbreviations are used in this manuscript:

VTE	venous thromboembolism
CAT	cancer-associated thromboembolism
PE	pulmonary thromboembolism
PESI	pulmonary embolism severity index
sPESI	simplified PESI
CT	computed tomography
CTPA	computed tomography-pulmonary angiography
KRS	Khorana Risk Score
RV/LV	right ventricle to left ventricle diameter ratio
ESC	European Society of Cardiology
ERS	European Respiratory Society
SI	shock index
PA	pulmonary artery
BMI	body mass index

## References

[1] Konstantinides S.V., Meyer G., Becattini C., Bueno H., Geersing G.J., Harjola V.-P., Huisman M.V., Humbert M., Jennings C.S., Jiménez D. (2020). 2019 ESC Guidelines for the diagnosis and management of acute pulmonary embolism developed in collaboration with the European Respiratory Society (ERS). Eur. Heart J..

[2] Creager M.A., Barnes G.D., Giri J., Mukherjee D., Jones W.S., Burnett A.E., Carman T., Casanegra A.I., Castellucci L.A., Clark S.M. (2026). 2026 AHA/ACC/ACCP/ACEP/CHEST/SCAI/SHM/SIR/SVM/SVN Guideline for the Evaluation and Management of Acute Pulmonary Embolism in Adults: A Report of the American College of Cardiology/American Heart Association Joint Committee on Clinical Practice Guidelines. J. Am. Coll. Cardiol..

[3] Heit J.A., Spencer F.A., White R.H. (2016). The epidemiology of venous thromboembolism. J. Thromb. Thrombolysis.

[4] Khan F., Tritschler T., Kahn S.R., Rodger M.A. (2021). Venous thromboembolism. Lancet.

[5] Falanga A., Schieppati F., Russo L., Soff G. (2019). Pathophysiology 1. Mechanisms of Thrombosis in Cancer Patients. Thrombosis and Hemostasis in Cancer; Cancer Treatment and Research.

[6] Geddings J.E., Mackman N. (2013). Tumor-derived tissue factor-positive microparticles and venous thrombosis in cancer patients. Blood.

[7] Nasser N.J., Fox J., Agbarya A. (2020). Potential Mechanisms of Cancer-Related Hypercoagulability. Cancers.

[8] Gussoni G., Frasson S., La Regina M., Di Micco P., Monreal M. (2013). Three-month mortality rate and clinical predictors in patients with venous thromboembolism and cancer. Findings from the RIETE registry. Thromb. Res..

[9] Khorana A.A., Mackman N., Falanga A., Pabinger I., Noble S., Ageno W., Moik F., Lee A.Y.Y. (2022). Cancer-associated venous thromboembolism. Nat. Rev. Dis. Prim..

[10] Khorana A.A., Francis C.W., Culakova E., Kuderer N.M., Lyman G.H. (2007). Thromboembolism is a leading cause of death in cancer patients receiving outpatient chemotherapy. J. Thromb. Haemost..

[11] Faiz A.S., Khan I., Beckman M.G., Bockenstedt P., Heit J.A., Kulkarni R., Manco-Johnson M., Moll S., Ortel T.L., Philipp C.S. (2015). Characteristics and Risk Factors of Cancer Associated Venous Thromboembolism. Thromb. Res..

[12] Donzé J., Le Gal G., Fine M.J., Roy P.-M., Sanchez O., Verschuren F., Cornuz J., Meyer G., Perrier A., Righini M. (2008). Prospective validation of the Pulmonary Embolism Severity Index. A clinical prognostic model for pulmonary embolism. Thromb. Haemost..

[13] Jiménez D., Aujesky D., Moores L., Gómez V., Lobo J.L., Uresandi F., Otero R., Monreal M., Muriel A., Yusen R.D. (2010). Simplification of the pulmonary embolism severity index for prognostication in patients with acute symptomatic pulmonary embolism. Arch. Intern. Med..

[14] Becattini C., Agnelli G., Vedovati M.C., Pruszczyk P., Casazza F., Grifoni S., Salvi A., Bianchi M., Douma R., Konstantinides S. (2011). Multidetector computed tomography for acute pulmonary embolism: Diagnosis and risk stratification in a single test. Eur. Heart J..

[15] Meinel F.G., Nance J.W., Schoepf U.J., Hoffmann V.S., Thierfelder K.M., Costello P., Goldhaber S.Z., Bamberg F. (2015). Predictive Value of Computed Tomography in Acute Pulmonary Embolism: Systematic Review and Meta-analysis. Am. J. Med..

[16] Sanchez O., Trinquart L., Colombet I., Durieux P., Huisman M.V., Chatellier G., Meyer G. (2008). Prognostic value of right ventricular dysfunction in patients with haemodynamically stable pulmonary embolism: A systematic review. Eur. Heart J..

[17] Khorana A.A., DeSancho M.T., Liebman H., Rosovsky R., Connors J.M., Zwicker J. (2021). Prediction and Prevention of Cancer-Associated Thromboembolism. Oncologist.

[18] Aujesky D., Perrier A., Roy P., Stone R.A., Cornuz J., Meyer G., Obrosky D.S., Fine M.J. (2007). Validation of a clinical prognostic model to identify low-risk patients with pulmonary embolism. J. Intern. Med..

[19] Goldstraw P., Chansky K., Crowley J., Rami-Porta R., Asamura H., Eberhardt W.E., Nicholson A.G., Groome P., Mitchell A., Bolejack V. (2016). The IASLC Lung Cancer Staging Project: Proposals for Revision of the TNM Stage Groupings in the Forthcoming (Eighth) Edition of the TNM Classification for Lung Cancer. J. Thorac. Oncol..

[20] Khorana A.A., Kuderer N.M., Culakova E., Lyman G.H., Francis C.W. (2008). Development and validation of a predictive model for chemotherapy-associated thrombosis. Blood.

[21] Cockcroft D.W., Gault M.H. (1976). Prediction of creatinine clearance from serum creatinine. Nephron.

[22] Cho S.U., Cho Y.-D., Choi S.-H., Yoon Y.-H., Park J.-H., Park S.-J., Lee E.-S. (2020). Assessing the severity of pulmonary embolism among patients in the emergency department: Utility of RV/LV diameter ratio. PLoS ONE.

[23] Remy-Jardin M., Ryerson C.J., Schiebler M.L., Leung A.N., Wild J.M., Hoeper M.M., Alderson P.O., Goodman L.R., Mayo J., Haramati L.B. (2021). Imaging of pulmonary hypertension in adults: A position paper from the Fleischner Society. Eur. Respir. J..

[24] Schulman S., Kearon C., Subcommittee on Control of Anticoagulation of the Scientific and Standardization Committee of the International Society on Thrombosis and Haemostasis (2005). Definition of major bleeding in clinical investigations of antihemostatic medicinal products in non-surgical patients. J. Thromb. Haemost..

[25] Li Y., Shang Y., Wang W., Ning S., Chen H. (2018). Lung Cancer and Pulmonary Embolism: What Is the Relationship? A Review. J. Cancer.

[26] Allara E., Shi W., Bolton T., Chalmers F., Brizzi L.F., Musto L., Shah A.S.V., Tomlinson C., Walter I., Conrad N. (2025). Burden of cardiovascular diseases in England (2020-24): A national cohort using electronic health records data. Lancet Public Health.

[27] Anderson F.A., Spencer F.A. (2003). Risk factors for venous thromboembolism. Circulation.

[28] Chew H.K., Wun T., Harvey D., Zhou H., White R.H. (2006). Incidence of venous thromboembolism and its effect on survival among patients with common cancers. Arch. Intern. Med..

[29] Becattini C., Maraziti G., Vinson D.R., Ng A.C.C., Exter P.L.D., Côté B., Vanni S., Doukky R., Khemasuwan D., Weekes A.J. (2021). Right ventricle assessment in patients with pulmonary embolism at low risk for death based on clinical models: An individual patient data meta-analysis. Eur. Heart J..

[30] Mansfield A.S., Tafur A.J., Wang C.E., Kourelis T.V., Wysokinska E.M., Yang P. (2016). Predictors of active cancer thromboembolic outcomes: Validation of the Khorana score among patients with lung cancer. J. Thromb. Haemost..

[31] Vathiotis I., Dimakakos E.P., Boura P., Ntineri A., Charpidou A., Gerotziafas G., Syrigos K. (2018). Khorana Score: Νew Predictor of Early Mortality in Patients With Lung Adenocarcinoma. Clin. Appl. Thromb. Hemost..

